# Non-Syndromic Hearing Impairment in India: High Allelic Heterogeneity among Mutations in *TMPRSS3*, *TMC1*, *USHIC*, *CDH23* and *TMIE*

**DOI:** 10.1371/journal.pone.0084773

**Published:** 2014-01-08

**Authors:** Aparna Ganapathy, Nishtha Pandey, C. R. Srikumari Srisailapathy, Rajeev Jalvi, Vikas Malhotra, Mohan Venkatappa, Arunima Chatterjee, Meenakshi Sharma, Rekha Santhanam, Shelly Chadha, Arabandi Ramesh, Arun K. Agarwal, Raghunath R. Rangasayee, Anuranjan Anand

**Affiliations:** 1 Molecular Biology and Genetics Unit, Jawaharlal Nehru Centre for Advanced Scientific Research, Bangalore, India; 2 Department of Genetics, Dr. ALM Post Graduate Institute of Basic Medical Sciences, Chennai, India; 3 Department of Audiology, Ali Yavar Jung National Institute for the Hearing Handicapped, Mumbai, India; 4 Department of ENT, Maulana Azad Medical College, New Delhi, India; Deutsches Krebsforschungszentrum, Germany

## Abstract

Mutations in the autosomal genes *TMPRSS3*, *TMC1*, *USHIC*, *CDH23* and *TMIE* are known to cause hereditary hearing loss. To study the contribution of these genes to autosomal recessive, non-syndromic hearing loss (ARNSHL) in India, we examined 374 families with the disorder to identify potential mutations. We found four mutations in *TMPRSS3*, eight in *TMC1*, ten in *USHIC*, eight in *CDH23* and three in *TMIE*. Of the 33 potentially pathogenic variants identified in these genes, 23 were new and the remaining have been previously reported. Collectively, mutations in these five genes contribute to about one-tenth of ARNSHL among the families examined. New mutations detected in this study extend the allelic heterogeneity of the genes and provide several additional variants for structure-function correlation studies. These findings have implications for early DNA-based detection of deafness and genetic counseling of affected families in the Indian subcontinent.

## Introduction

Hearing impairment is the most common sensory defect in humans, occurring at a frequency of about one in 1000 live births, of which 50% are due to genetic causes [1]. About 70% of hereditary hearing loss is non-syndromic, wherein hearing impairment is not associated with any additional clinical phenotype. To date, 65 genes for non-syndromic hearing loss (NSHL) have been identified (http://hereditaryhearingloss.org/) [2]. Mutations in *TMPRSS3* (transmembrane serine protease 3) [3], *TMC1* (transmembrane cochlear-expressed gene 1) [4], *USHIC* (Usher 1C) [5], *CDH23* (cadherin 23) [6] and *TMIE* (transmembrane inner ear) [7] are known to play a causative role in NSHL. Indeed, *TMPRSS3* [3], *TMC1* [4] and *TMIE* [7], were identified in studies involving a few multi-affected families from the Indian subcontinent. However, a detailed study evaluating the contribution of these genes has not been carried out for Indian populations. In this study, we describe the spectrum of mutations in *TMPRSS3*, *TMC1*, *USHIC*, *CDH23* and *TMIE* in 374 families with ARNSHL from India.

## Materials and Methods

### Subjects

A total of 1739 individuals from 374 families with at least two members affected with recessive, prelingual, severe-to-profound NSHL were included in this study. Of these 316 were families with two affected sibs, 54 with three affected sibs and 4 with four or more affected sibs. These families had been ruled out for mutations in *Cx26* (connexin 26, *GJB2*), which is known to be the most common cause of hereditary hearing loss in India [8]. A detailed clinical history of each affected member was collected to ensure that hearing loss was not due to infections, ototoxic drugs, trauma or premature birth and was not accompanied by any apparent ear, eye, head, neck, skin, skeletal or neurological abnormalities. The degree of hearing loss was ascertained by audiological evaluation involving pure tone audiometry, which included bone conduction. Hearing thresholds were obtained between 250–8000 Hz in a sound-treated room. Ten milliliters of venous blood was collected from members of the families. Fifty-four healthy unaffected individuals, above 20 years of age without any apparent family history of hearing impairment, were also included in this study as controls to estimate allele frequencies of the sequence variants found during the course of this work. Genomic DNA was extracted using the phenol-chloroform method [9]. This study was approved by the Institutional Human Bioethics and Biosafety Committees of the four institutes involved in the work and informed written consent was obtained from all participating individuals and from the parents of those affected individuals who were younger than 18 years of age.

### Genetic analysis

To identify families that may harbour mutations in *TMPRSS3*, *TMC1*, *USHIC* and *CDH23*, we carried out concordance/discordance tests using polymorphic microsatellite markers flanking the genes (Table 1) in 374 families. These markers were amplified using the polymerase chain reaction (PCR) with 50 ng of genomic DNA, 25 pmol primers, 1.5 mM MgCl_2_ and 2.5 U *Taq* DNA polymerase. PCR was carried out using a GeneAmp PCR 9700 and genotyping using an ABI PRISM 3730 DNA Analyzer (Applied Biosystems, USA). Allele sizing was done using GENEMAPPER v3.7 (Applied Biosystems). For the families that could not be excluded on the basis of marker discordance among affected siblings, complete *TMPRSS3* (13 exons), *TMC1* (24 exons), *USH1C* (28 exons) and *CDH23* (70 exons) transcript structures, comprising exonic and flanking intronic regions, were analyzed by direct sequencing. For *TMIE* (DFNB6), direct sequencing of its four exons and flanking intronic regions for an affected member, in each of the 374 families, was carried out. The primers for sequencing were designed using PRIMER3 (http://primer3.ut.ee) [10]. PCR was performed and the amplified products were purified by Montage PCR96 Cleanup reagents (Millipore). Cycle sequencing was performed using 20 ng of purified PCR products, 3.2 pmol of each primer and ABI PRISM BigDye Terminator cycle sequencing reagents. Following cycle sequencing, the samples were loaded onto an ABI PRISM 3730 DNA Analyzer. Each amplicon was sequenced in both directions and analyzed using DNASTAR SeqmanII 5.01.

**Table 1:** Genes and locations of microsatellite markers used in the concordance/discordance tests.

Gene (locus)	Polymorphic microsatellite markers and their locations
*TMPRSS3* (DFNB8/10)	D21S1260 (900 kb centromeric), D21S1225 (128 kb centromeric), D21S49 (83 kb telomeric) and D21S1411 (344 kb telomeric)
*TMC1* (DFNB7/11, DFNA36)	D9S789 (1.3 Mb centromeric), D9S1822 (300 kb telomeric) and D9S1876 (an intragenic marker).
*USH1C* (DFNB18)	D11S902 (25 kb telomeric), D11S4130 (180 kb centromeric), D11S1888, (190 kb centromeric) and D11S4138 (175 kb centromeric)
*CDH23* (DFNB12)	D10S537 (1 Mb centromeric), D10S1688 (860 kb centromeric), D10S412 (147 kb centromeric) and D10S218 (300 kb telomeric).

### Bioinformatic analysis

The amino acid and nucleotide residue conservations across species were examined using NCBI BLAST (http://www.ncbi.nlm.nih.gov/BLAST/) and conservation across protein or gene families using ClustalW (http://www.ebi.ac.uk/clustalw/) [11] and ConSeq (consurf.tau.ac.il/) [12]. Splice site prediction was done using NetGene2 (http://www.cbs.dtu.dk/services/NetGene2) [13]; disulphide bond prediction, using DISULFIND (http://disulfind.dsi.unifi.it/) [14]. The possible pathogenic effect of protein-coding variants was examined using two prediction tools: SIFT (http://sift.jcvi.org/) [15] and Polyphen-2 (http://genetics.bwh.harvard.edu/pph2/) [16].

## Results

### Pathogenic and apparently benign variants in *TMPRSS3*, *TMC1*, *CDH23*, *USH1C* and *TMIE*

**Figure 1:**
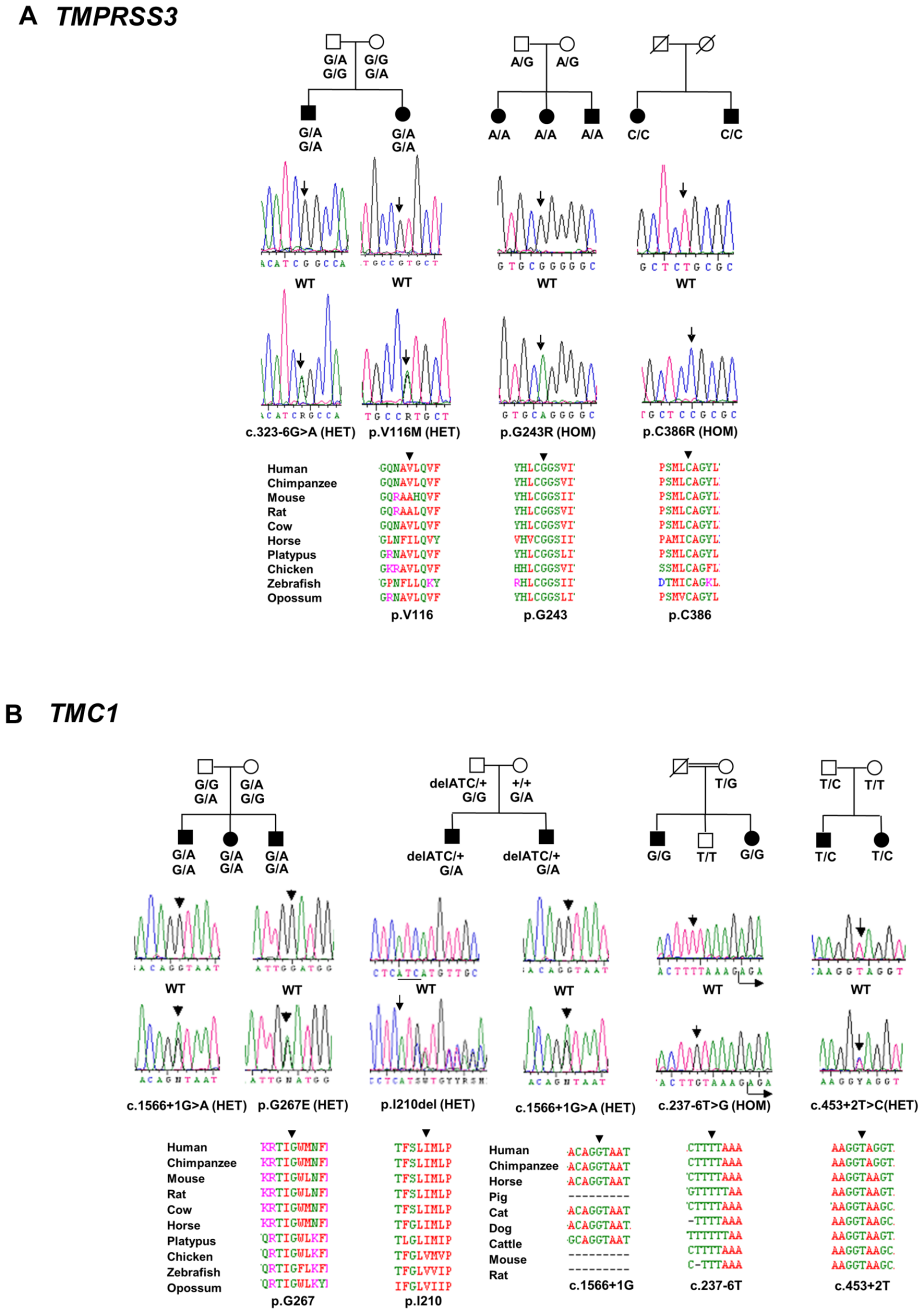
Analysis of segregation and conservation of novel variants in *TMPRSS3* and *TMC1*. A) *TMPRSS3* and B) *TMC1*. Top panel shows the family structure and segregation of the variants; in cases where the variants were seen in more than one family, a single representation is provided; middle panel shows the electropherogram and lower panel shows the conservation of the mutated residue.

**Figure 2:**
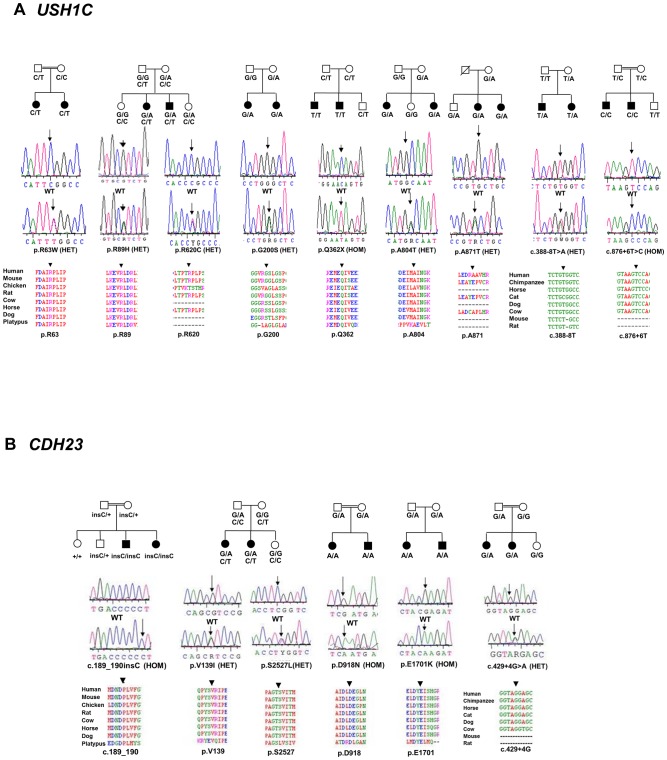
Analysis of segregation and conservation of novel variants in *USH1C* and *CDH23*. A) *USHIC* and B) *CDH23*. Top panel shows the family structure and segregation of the variants; in cases where the variants were seen in more than one family, a single representation is provided; middle panel shows the electropherogram and lower panel shows the conservation of the mutated residue.

For the aforementioned genes, concordance/discordance test were carried out in the 374 ARNSHL families. Based on this test, the possibility for mutation could not be excluded in 48 families for *TMPRSS3*, in 50 families for *TMC1*, in 24 families for *CDH23* and in 46 families for *USH1C*. Further analysis of one affected member in each of the families revealed 12 variants in *TMPRSS3*, 20 in *TMC1*, 36 in *USH1C* and 44 in *CDH23*. In *TMIE*, 11 variants were identified. To assess their pathological potential, these variants were evaluated for (i) segregation among additional affected members of the families, (ii) evolutionary conservation of nucleotide or amino acid residue and (iii) frequency among individuals with apparently normal hearing. These criteria helped identify 33 potential mutations: four in *TMPRSS3*, eight in *TMC1*, ten in *USH1C*, eight in *CDH23* and three in *TMIE* (Table 2, Figure 1 and 2). Additionally, 90 apparently benign gene variants were found, which included 77 known polymorphisms (http://www.ncbi.nlm.nih.gov/) and 13 new ones. Of the new ones, five were in *TMC1*, two in *USH1C*, two in *CDH23* and four in *TMIE*. The variants classified as benign satisfied one or more of the following criteria: (i) allele frequency of 0.01 or more among the control individuals; (ii) lack of segregation with the deafness phenotype; (iii) poor evolutionary conservation and (iv) apparently no effect on transcript or protein function (Table S1).

**Table 2:** Mutations in the *TMPRSS3, TMC1, USH1C, CDH23* and *TMIE* genes.

Gene sequence variant	Location	Domain	Possible effect on gene or protein	SIFT	PolyPhen-2	Hom/Het	Number of families: samples per family	Novel or known	dbSNP Accession	Frequency in control chromosomes
**A. ** ***TMPRSS3***										
c.323-6G>A	Intron 4	-	Splice site regulation	-	-	Hom + ompound het	3+1∶4,4,3+4	Known[3]	-	-
c.346G>A	Exon 5	SRCR	p.V116M	Damaging	Probably damaging	Compound het	1∶4	Novel	rs200090033^a^	0/102
c.727G>A	Exon 8	Serine Protease	p.G243R	Damaging	Probably damaging	Hom	1∶5	Novel	-	0/108
c.1156T>C	Exon 11	Serine Protease	p.C386R	Damaging	Probably damaging	Hom	1∶2	Novel	-	0/108
**B. ** ***TMC1***										
c.100C>T	Exon 7	N-TERM	p.R34X	-	-	Hom	1∶4	Known[4]	rs121908073^a^	-
c.237-6T>G	Intron 7	-	Splice site regulation	-	-	Hom	1∶4	Novel	-	0/100
c.453+2T>C^b^	Intron 9	-	Splice site regulation	-	-	Het	1∶4	Novel	-	0/102
c.628_630del	Exon 11	TM1	p.I210del	-	-	Compound het	1∶4	Novel	-	0/102
c.800G>A	Exon 13	EC1-LOOP	p.G267E	Tolerated	Probably damaging	Compound het	1∶5	Novel	-	0/108
c.1114G>A	Exon 15	TM3	p.V372M	Damaging	Probably damaging	Hom	2∶6,6	Known[26]	-	-
c.1333C>T	Exon 16	TM4	p.R445C	Damaging	Probably damaging	Hom	1∶2	Known[27]	-	0/108
c.1566+1G>A	Intron 17	-	Splice site regulation	-	-	Compound het	2∶4,5	Novel	-	0/106
**C. ** ***USH1C***										
c.187C>T^b^	Exon 3	N-TERM	p.R63W	Damaging	Probably damaging	Het	1∶4	Novel	-	0/100
c.267G>A	Exon 4	N-TERM	p.R89H	Damaging	Probably damaging	Compound het	1∶6	Novel	-	0/100
c.388-8T>A^b^	Intron 4	-	Splice site regulation	-	-	Het	1∶4	Novel	-	0/100
c.496+1G>A	Intron 5	-	Splice site regulation	-	-	Hom	1∶5	Known[20]	-	0/100
c.598G>A^b^	Exon 8	Proximal to PDZ	p.G200S	Tolerated	Benign	Het	1∶4	Novel	-	0/100
c.876+6T>C	Intron 11	-	Splice site regulation	-	-	Hom	1∶5	Novel	-	0/100
c.1084C>T	Exon 13	CC1	p.Q362X	-	-	Hom	1∶5	Novel	-	0/100
c.1858C>T	Exon 19	PST	p.R620C	Damaging	Probably damaging	Compound het	1∶6	Novel	rs143160805^a^	0/100
c.2410G>A^b^	Exon 25	PDZ3	p.A804T	Tolerated	Probably damaging	Het	1∶5	Novel	rs 150593932^a^	0/100
c.2611G>A^b^	Exon 27	C-TERM	p.A871T	Tolerated	Benign	Het	1∶4	Novel	rs56165709^a^	0/100
**D. ** ***CDH23***										
c.189_190insC	Exon 4	EC1	Frameshift	-	-	Hom	2∶6,5	Novel	-	0/96
c.415G>A	Exon 6	Between EC1 and EC2	p.V139I	Tolerated	Benign	Compound het	1∶5	Novel	-	0/96
c.429+4G>A^b^	Intron 6	-	Splice site regulation	-	-	Het	1∶5	Novel	-	0/96
c.2752G>A	Exon 25	EC9	p.D918N	Damaging	Probably damaging	Hom	1∶4	Novel	-	0/96
c.2968G>A	Exon 26	EC9	p.D990N	Tolerated	Probably damaging	Hom	1∶4	Known[28]	-	-
c.5101G>A	Exon 40	EC16	p.E1701K	Damaging	Probably damaging	Hom	1∶4	Novel	-	0/96
c.5660C>T^b^	Exon 43	EC18	p.T1887I	Tolerated	Probably damaging	Het	2∶4,3	Known[29]	-	0/96
c.7580C>T	Exon 54	EC24 proximal	p.S2527L	Damaging	Probably damaging	Compound het	1∶5	Novel	-	0/96
**E. ** ***TMIE***										
c.92A>G	Exon 1	EC-LOOP	p.E31G	Damaging	Probably damaging	Hom	2∶4,5	Known[30]	-	-
c.125_126insCGCC	Exon 2	EC-LOOP	Frameshift	-	-	Hom	4∶6,6, 4,5	Known[7]	-	-
c.250C>T	Exon 3	C-TERM	p.R84W	Damaging	Probably damaging	Hom	2∶5,6	Known[7]	rs28942097	-

^a^ Allele frequency in the range of 0.000– 0.003 is observed for the variant in the dbSNP137 database. Gene sequence variants shown are either almost certainly pathogenic alleles, or ^b^potentially pathogenic. CC: coiled-coiled, C-TERM: C-Terminal, EC-LOOP: Extracellular loop, EC: Cadherin extracellular repeat domain, Hom: Homozygous, Het: Heterozygous, N-TERM: N-Terminal, PDZ: Post synaptic density protein-95, Drosophila disc large tumor suppressor, Zonula occludens-1 domain, PST: Proline-serine-threonine rich region, SRCR: scavenger receptor cysteine-rich, TM: Transmembrane domain.

*TMPRSS3*: Gene ID: 64699; mRNA: NM_024022; Protein: NP_076927

*TMC1*: Gene ID: 117531; mRNA: NM_138691; Protein; NP_619636

*USH1C*: Gene ID: 10083; mRNA: NM_153676; Protein: NP_710142

*CDH23*: Gene ID: 64072; mRNA: NM_022124.3; Protein: NP_071407.3

*TMIE*: Gene ID: 259236; mRNA: NM_147196.1; Protein: NP_671729.

### Exonic mutations

**Figure 3:**
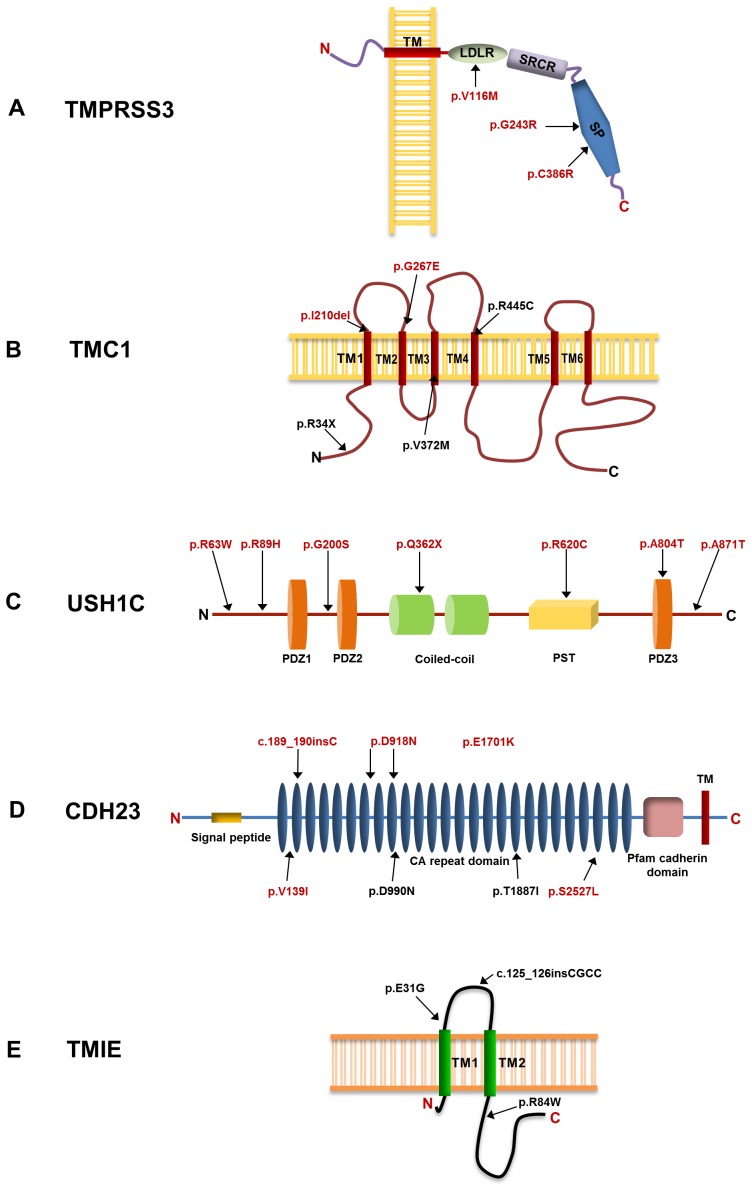
Schematic representation of TMPRSS3, TMC1, USH1C, CDH23 and TMIE. A) TMPRSS3, B) TMC1, C) USH1C, D) CDH23 and E) TMIE. Arrows point to the location of the mutations. Shown in red are the mutations identified in the study.

Among the mutations likely to affect protein structure or function were the nonsense mutations: p.R34X in TMC1 and p.Q362X in USH1C and, 20 missense mutations: p.V116M, p.G243R and p.C386R in TMPRSS3; p.G267E, p.V372M and p.R445C in TMC1; p.R63W, p.R89H, p.G200S, p.R620C, p.A804T and p.A871T in USH1C; p.V139I, p.D918N, p.D990N, p.E1701K, p.T1887I and p.S2527L in CDH23; p.E31G and p.R84W in TMIE. In addition, a deletion, p.I210del in TMC1 and two insertions: CDH23, c.189_190insC and TMIE, c.125_126insCGCC were also identified. Several of the substitution mutations (p.V116M, p.G243R, p.C386R, p.V372M, p.R445C, p.R63W, p.R89H, p.R620C, p.D918N, p.E1701K, p.S2527L, p.E31G and p.R84W) were predicted to have severe detrimental effects by SIFT and POLYPHEN analysis (Table 2). Further, many of the identified coding mutations (p.V116M, p.G243R, p.C386R, p.I210del, p.V372M, p.R445C, p.R620C, p.A804T, p.V139I, p.D918N, p.D990N, p.E1701N and p.T1887I) reside in structurally or functionally important protein domains (Table 2, Figure 3). Upon sequence comparison across species and protein families, a high degree of evolutionary-conservation was observed for the amino acid residues at the mutation sites (Figure 1 and 2). For example, in TMPRSS3, p.G243R and p.C386R mutations occur in the highly conserved catalytic serine protease domain. Disulfide bond prediction and available crystal structure of the extracellular region of Hepsin (TMPRSS1, pdb 1z8g) suggest the presence of a disulfide bridge involving Cys386 and Cys370 in TMPRSS3, similar to the disulphide bond of corresponding Cys338 and Cys322 in Hepsin [17]. Mutation p.V116M, is predicted to be a damaging substitution in the SRCR domain involved in binding of TMPRSS3 to the cell surface and in its interaction with the extracellular molecules [18]. In p.I210del, conservation analysis by ConSeq, showed that the deleted isoleucine is located in a stretch of conserved and buried hydrophobic residues of the first transmembrane domain of TMC1. Mutation p.A804T resides in the third PDZ domain and, mutation p.A871T in the C-terminal tail of USH1C. p.D918N and p.E1701K in CDH23, disrupt the highly conserved peptide motifs, DXD and LDRE, respectively [19]. These motifs are involved in binding of calcium ions for the interdomain rigidification of the cadherin repeat domains. The c.189_190insC insertion mutation causes a frameshift leading to premature termination after an incorporation of 19 unrelated amino acids in CDH23.

### Intronic mutations

The following novel intronic mutations were observed: c.237-6T>G, c.453+2T>C and c.1566+1G>A in *TMC1*; c.388-8T>A and c.876+6T>C in *USHIC* and c.429+4G>A in *CDH23*. Potential effects of the intronic variants on regulation of splicing were predicted by using NetGene2 and by examining evolutionary conservation of the nucleotide residues. c.453+2T>C, c.1566+1G>A and c.876+6T>C are predicted to affect the 5′ splice-site donor usage. c.237-6T>G is a change in the conserved polypyrimidine tract flanking 3′ splice acceptor site in intron 7. c.388-8T>A could generate a relatively strong splice acceptor site, which would introduce two additional residues, valine and lysine, at positions 129 and 130 in the conserved stretch of first PDZ domain. *TMPRSS3*, c.323-6G>A [3] and *USH1C*, c.496+1G>A are previously known splice-site mutation observed in this study [20].

### Homo-, hetero- and compound hetero- zygotes, and a search of *CDH23* and *USH1C* interacting alleles

In five families with *TMPRSS3* mutations, all affected members were homozygotes and in one family all affected members were compound heterozygotes (p.V116M and c.323-6G>A) (Table 2). In five families with *TMC1* mutations, all affected members were homozygotes, whereas in two families affected members were compound heterozygotes (c.1566+1G>A/p.I210del and c.1566+1G>A/p.G267E). In one family with a *TMC1* mutation, heterozygotes were affected. For *USH1C* mutations, affected individuals were homozygotes in three families, compound heterozygotes (p.R620C/p.R89H) in one family and heterozygotes in five families. In case of *CDH23* mutations, affected members in five families were homozygotes; in three families they were heterozygotes and in one family they were compound heterozygotes (p.V139I/p.S2527L). For *TMIE*, all eight mutation positive families were homozygous for the observed mutations. Among the 40 families that were mutation positive, 26 were homozygous for the mutation; five, compound heterozygous and in nine families, the mutation occurred in a heterozygous condition. For five *USHIC* and three *CDH23* families carrying heterozygous potentially pathogenic alleles, we examined sequences of the genes known to interact with *USHIC* and *CDH23*, for the possibility of the presence of a second mutation. Families carrying heterozygous variants in *USHIC* (Table 2C) were analyzed for the *MYO7A* [21], *CDH23* [6] and *SANS* [22] genes. Similarly, *USH1C*, which is known to interact with *CDH23*, was analyzed in the three families carrying heterozygous changes in *CDH23* (Table 2D). In the five *USHIC* families studied, six new changes (p.V66V, p.R412H, p.A1425V, p.N1667K, c.9510+13C>T and p.D3253A) were observed as heterozygous variants in addition to several known polymorphisms in *CDH23*. p.R412H, p.A1425V and p.N1667K did not segregate with the phenotype in the families studied; and p.V66V, c.9510+13C>T and p.D3253A were present in control chromosomes of unaffected individuals, implying that these changes were unlikely to be pathogenic. In an analysis of 48 exons of *MYO7A* in the families heterozygous for *USH1C* mutations, one new intronic variant c.736-73C>T was observed in four out of the five families examined. This change did not segregate with the phenotype. No variant was observed in the three exons of *SANS*. Similarly, no novel potentially pathogenic *USHIC* alleles were detected, in the three families with the heterozygous *CDH23* variants.

## Discussion

The contribution of mutations to ARNSHL has been found to be variable among populations from different parts of the world. Population-specific social parameters such as marriage among persons with hearing loss and consanguinity may affect the prevalence of a mutation, leading to certain mutations becoming common in one population and rare in another. Therefore, population-specific studies are necessary to understand the contribution of mutations to the genetic load. Studies carried out earlier on Indian families with ARNSHL have revealed that mutations in *Cx26* are the most common cause of the disorder and account for about 25% of severe-to-profound hereditary hearing loss in India [8]. However, data on families from the Indian subcontinent have not been available previously for the genes examined in this study.

In TMPRSS3 three novel mutations, p.G243R, p.C386R and p.V116M, were detected. As mentioned earlier, Gly243 and Cys386 are located in the highly conserved catalytic serine protease domain of the protein. Glycine is a small amino acid, usually known to play a crucial role in protein structure. In p.G243R, the uncharged glycine is substituted by a large polar residue, arginine, which may affect protein-folding and, therefore, TMPRSS3 function. Six conserved cysteine residues, C242, C258, C370, C386, C397 and C425, present in the serine protease domain of TMPRSS3, are likely to form intra subunit disulfide bonds [17]. p.C386R is possibly altering the secondary structure of the serine protease domain of TMPRSS3 protein and affecting its function. In TMC1, nonsense mutation, p.R34X is known to occur at a high frequency in Pakistan and may be due to a founder effect: an SNP marker c.45C>T (rs2589615) was observed in all the families with p.R34X from Pakistan [23]. Interestingly, in this study c.45C>T was also observed in the family in which p.R34X was found. The age of this mutation has been estimated to be between 1075 and 1900 years [24]. In *TMC1*, two mutations, c.237-6T>G and c.453+2T>C, are likely to affect splicing. p.I210del in TMC1 occurs in the first transmembrane domain. Deletion of isoleucine in the conserved region of the first transmembrane domain might affect its topology. A highly conserved uncharged glycine is mutated to an acidic amino acid glutamic acid in the extracellular region of TMC1 in p.G267E, which is likely to affect protein structure. Two intronic mutations, c.1566+1G>A and c.453+2T>C, in *TMC1* are proposed to affect splicing, leading to a frameshift and formation of a non-functional protein. In *TMC1*, c.237-6T>G, a transition of thiamine to guanine, 4 bases before the splice acceptor site in intron 7, is likely to affect the polypyrimidine tract. The polypyrimidine tract is one of the *cis*-acting elements in the splicing machinery that is recognized by several protein factors to form a functional spliceosome [25]. In CDH23, five out of the eight mutations reside in the calcium-binding EC domains. EC domains are thought to have a critical role in rigidification, linearization and dimerization of cadherin proteins.

A total of 123 variants were observed in this study, of which 10 are known deafness mutations, 23 are previously unreported mutations, and 90, apparently neutral variants. Further, functional validation of the variants identified in this study is likely to result in a better understanding of their pathogenic potential. Before sequencing transcript structures of the *TMC1*, *TMPRSS3*, *CDH23* and *USH1C* genes, we examined concordance/discordance of the microsatellite markers tightly linked to the gene of interest. Those families which showed a clear discordance of the markers among affected siblings were excluded from further analysis of the gene. However, families which showed marker-concordance as well as the ones which were uninformative for the markers, were examined for mutations by sequencing all known exonic and flanking intronic regions of the genes. We may have missed a mutation due to an intragenic recombination event, genetic heterogeneity, or when the mutation is present in deep intronic regions or *cis*-regulatory regions of the gene. Large exonic deletions or in/dels as well as those second site mutations which occur in heterozygous carriers may have also gone undetected.

Among the sequence variants found in this study, eight were found to occur in a heterozygous condition. These were rare variants and were present at conserved locations. Five of these were in *USHIC*, two in *CDH23* and one in *TMC1* (Table 2). These changes seem potentially pathogenic. It is possible that individuals with these variants are hearing impaired due to another unknown mutation at these genes or another gene.

Mutational survey of *TMPRSS3*, *TMC1*, *USH1C*, *CDH23* and *TMIE* genes have been carried out in certain world populations. These data suggest that the contribution of these genes to ARNSHL is not the same in all population and varies from 0.5-5% (Table 3). Our observations suggest that the overall contribution of *TMPRSS3*, *TMC1, USH1C, CDH23* and *TMIE* mutations for ARNSHL is low in India: 1.2% of the hearing impaired examined showed mutations in *TMPRSS3*, 1.6% in *TMC1*, 1.8% in *USH1C*, 1.8% in *CDH23* and 1.6% in *TMIE*. Unlike mutations in the *Cx26* gene, which are the most common cause of hereditary impairment in India, the contribution of mutations in these five genes is rather small. The spectra of alleles in the *TMPRSS3, TMC1, USH1C* and *CDH23* genes in Indian populations seem to be quite different from those observed for other world populations; among the 33 mutations observed in our study 23 were not reported in other populations. These studies have implications for early detection of hearing loss, genetic counseling, and for implementation of suitable early intervention strategies.

**Table 3:** Genetic epidemiology of the *TMPRSS3, TMC1, USH1C, CDH23* and *TMIE* genes.

Population	Subjects	Prevalence/Mutation Positives	Reference
***TMPRSS3***			
Caucasian	448 NSHL probands negative for *Cx26*, 35delG	0.45%	[31]
Pakistan	159 NSHL families, 449 ARNSHL families, 353 ARNSHL families	2.5%, 1.8%, 10 families	[32], [33], [34]
Turkey	49 NSHL families negative for *Cx26* two *Cx30* genomic deletions and a mitochondrial mutation in *MTRNR1*, 1555A>G, 86 ARNSHL families negative for *Cx26*, 25 ARNSHL families	1.7%, 8%	[35], [36]
Tunisia	39 ARNSHL families	2 families	[37]
Korea	40 ARNSHL subjects	2.5%	[38]
India	374 ARNSHL families negative for *Cx26*	1.2%	This study
***TMC1***			
Pakistan and India	230 ARNSHL families, 168 ARNSHL families negative for *Cx26*, 557 ARNSHL large families	5.4%, 4.4%, 3.4%	[4], [26], [23]
Turkey	65 ARNSHL families negative for *Cx26*, 49 NSHL families negative for *Cx26*, two *Cx30* genomic deletions and a mitochondrial mutation in *MTRNR1*, 1555A>G, 86 ARNSHL families negative for *Cx26*	6%, 6.6%, 8.1%	[39], [27], [35]
Iran	54 ARNSHL families	1 family	[40]
India	374 ARNSHL families negative for *Cx26*	1.6%	This study
***USH1C***			
China	32 recessive NSHL families	1 family	[41]
Caucasian	16 NSHL sib pairs + 2 NSHL families	0%	[42]
***CDH23***			
America, Sweden, Dutch, German, Spain, Pakistan, South Africa, France, Italy, Ireland	38 recessive NSHL families negative for *Cx26* and *MYO7A*	5%	[29]
Japan	64 ARNSHL probands negative for *Cx26*, 919 probands from ARNSHL families	5%, 5.4%	[43], [44]
Turkey	49 NSHL families negative for *Cx26*, two *Cx30* genomic deletions and a mitochondrial mutation in *MTRNR1*, 1555A>G	3.3%	[35]
India	374 ARNSHL families negative for *Cx26*	1.8%	This study
***TMIE***			
Pakistan	168 ARNSHL families negative for *Cx26*	1.7%	[30]
Turkey	49 NSHL families negative for *Cx26*, two *Cx30* genomic deletions and a mitochondrial mutation in *MTRNR1*, 1555A>G	6.6%	[35]
Taiwan	250 NSHL subjects	1 subject	[45]
India	374 ARNSHL families negative for *Cx26*	1.6%	This study

## Supporting Information

Table S1 **New benign gene variants observed in** ***TMC1*** **,** ***USH1C*** **,** ***CDH23*** **and** ***TMIE*** **.**
(DOC)
