# Supplementary material for: Non-Syndromic Hearing Impairment in India: High Allelic Heterogeneity among Mutations in TMPRSS3, TMC1, USHIC, CDH23 and TMIE
Source: PLoS One. 2014 Jan 8;9(1):e84773. doi: 10.1371/journal.pone.0084773 (PMC3885616; doi:10.1371/journal.pone.0084773)
Supplement: Table S1 — New benign gene variants observed in TMC1 , USH1C , CDH23 and TMIE . (DOC) [file pone.0084773.s001.doc]

**Table S1.**  New benign gene variants observed in *TMC1, USH1C, CDH23* and *TMIE*.

| **Gene** | **Gene sequence variant** | **Location** | **Possible effect on protein** | **Allele frequency** |
| --- | --- | --- | --- | --- |
| *TMC1* | c.245_247del | protein-coding | p.E83del | 0.01 |
| *TMC1* | c.363-26C>A | intron | - | 0.02 |
| *TMC1* | c.462A>G | protein-coding | p.A154A | 0.02 |
| *TMC1* | c.1405-13C>G**^a^** | intron | - | 0.00 |
| *TMC1* | c.1566+4delA**^a^** | intron | - | 0.00 |
| *USH1C* | c.378C>T | protein-coding | p.V126V | 0.02 |
| *USH1C* | c.1113G>A | protein-coding | p.K381K | 0.00 |
| *CDH23* | c.3931G>C | protein-coding | p.V1211V | 0.19 |
| *CDH23* | c.2588-86C>A | intron | - | 0.13 |
| *TMIE* | c.1-95_-83del13bp**^b^** | 5’ UTR | - | 0.00 |
| *TMIE* | c.-49G>A**^c^** | 5' UTR | - | 0.00 |
| *TMIE* | c.-37C>A | 5' UTR | - | 0.02 |
| *TMIE* | c.148G>T | protein-coding | p.V50L | 0.01 |

**^a^** The intronic variants were predicted to have no effect on splice-site regulation and occurred in unconserved gene regions.

**^b^** This variant was observed in three families. In each of these families, unaffected parents and affected offsprings were heterozygous for the variation.

**^c^** The variant did not segregate with the clinical phenotype.
